# Linear Epitopes of *Paracoccidioides brasiliensis* and Other Fungal Agents of Human Systemic Mycoses As Vaccine Candidates

**DOI:** 10.3389/fimmu.2017.00224

**Published:** 2017-03-10

**Authors:** Luiz R. Travassos, Carlos P. Taborda

**Affiliations:** ^1^Department of Microbiology, Immunology and Parasitology, Federal University of São Paulo, São Paulo, Brazil; ^2^Department of Microbiology, Institute of Biomedical Sciences, University of São Paulo, São Paulo, Brazil; ^3^Laboratory of Medical Mycology IMTSP/LIM53/HCFMUSP, University of São Paulo, São Paulo, Brazil

**Keywords:** peptide, vaccine, antibody, fungi, *Paracoccidioides*

## Abstract

Dimorphic fungi are agents of systemic mycoses associated with significant morbidity and frequent lethality in the Americas. Among the pathogenic species are *Paracoccidioides brasiliensis* and *Paracoccidioides lutzii*, which predominate in South America; *Histoplasma capsulatum, Coccidioides posadasii*, and *Coccidioides immitis*, and the *Sporothrix* spp. complex are other important pathogens. Associated with dimorphic fungi other important infections are caused by yeast such as *Candida* spp. and *Cryptococcus* spp. or mold such as *Aspergillus* spp., which are also fungal agents of deadly infections. Nowadays, the actual tendency of therapy is the development of a pan-fungal vaccine. This is, however, not easy because of the complexity of eukaryotic cells and the particularities of different species and isolates. Albeit there are several experimental vaccines being studied, we will focus mainly on peptide vaccines or epitopes of T-cell receptors inducing protective fungal responses. These peptides can be carried by antibody inducing β-(1,3)-glucan oligo or polysaccharides, or be mixed with them for administration. The present review discusses the efficacy of linear peptide epitopes in the context of antifungal immunization and vaccine proposition.

## Systemic Fungal Infections and Current Treatment: A Short Introduction

Distinct groups of fungi can cause systemic mycoses: geographically delimitated thermal-dimorphic fungi, classical yeast such as *Cryptococcus* spp. and *Candida* spp., or molds like *Aspergillus* spp., *Fusarium* spp., and *Penicillium* spp.

Thermal-dimorphic fungi are a group of ascomycetes endemic in certain regions, agents of the most common diseases, such as paracoccidioidomycosis, occurring in the vast area from south Mexico to the north of Argentina; coccidioidomycosis in the Americas with particular incidence in the USA (California, Texas, Utah, New Mexico, Arizona, and Nevada), Mexico, Colombia, Venezuela, northeast of Brazil, and north of Argentina; North American blastomycosis, with high incidence in Canada, eastern USA, sporadic cases in Argentina, and endemic areas in middle and eastern Africa; histoplasmosis, found in the Americas, Southeast Asia, and Africa; and the *Sporothrix schenckii* complex with worldwide distribution (1). These fungi usually present propagules in the soil, vegetal, or animal excrement. The infection usually starts *via* the respiratory route except for sporotrichosis that rarely occurs by inhalation of fungal propagules, rather arising from surface injuries by fungus-contaminated objects or cat scratches (1).

Most of patients developing *Candida* spp. and *Cryptococcus* spp. infections are immunodeficient suffering from AIDS, diabetes, or have been administered immunosuppressive drugs as in organ transplantation procedures, indwelling catheter for a short or long time, although primary infections can also occur without association with other conditions (2, 3). *Aspergillus fumigatus, Fusarium* spp., and *Penicillium* spp. can cause different types of infection. Patients undergoing hematopoietic stem cell transplantation for treatment of hematological malignancy have considerable risk of developing fatal fungal infection (4, 5). Whereas infection by *Candida* spp. occurs mainly by endogenous yeast, this is not an exclusive pathway. Infections by *Cryptococcus* spp., *A. fumigatus, Fusarium* spp., and *Penicillium* spp. occur by inhalation of fungal propagules (2–5).

There is no trustworthy quantitation of people infected by systemic mycosis in the World; however, Brown et al. (6) estimated that more than 2,050,000 people yearly infected with the 10 most significant invasive fungal agents/mycoses including *Aspergillus, Candida, Cryptococcus*, mucormycosis, *Pneumocystis, Blastomyces, Coccidioides, Histoplasma, Penicillium*, and *Paracoccidioides* (6).

There are few groups of antifungal drugs effective in the treatment of systemic fungal disease. Most of them belong to four classes: polyenes, azoles, echinocandins, and pyrimidine (7). Other antimicrobial drugs also have antifungal action such as trimethoprim-sulfamethoxazole that is used with relative success in the treatment of patients with paracoccidioidomycosis (8). Treatment and the option for antifungal drugs depend on the severity of the disease and time of use (9).

There are many reports on drug resistance in systemic fungal infections involving almost all classes of antimicrobial drugs. In paracoccidioidomycosis, resistance to ketoconazole and trimethoprim-sulfamethoxazole may be related to the agent species (*Paracoccidioides brasiliensis* or *Paracoccidioides lutzii*) [reviewed in Ref. (9)] or the melanization process, which enhances the resistance of yeast cells to amphotericin B (10). The biofilm formation in *Candida* spp. can enhance resistance of yeast cells to antifungal drugs (11, 12), and the *Candida albicans* biofilm is intrinsically resistant to the host immune system [reviewed in Ref. (12)]. Such resistance appears to be multifactorial involving conventional resistance mechanisms as the increased efflux pump, and mechanisms specific to the biofilm as the production of an extracellular matrix containing β-glucan and extracelluar DNA [reviewed in Ref. (11)]. The resistance to azoles by efflux pump proteins in *Candida albicans* may involve overexpression of Cdr1p (ATP-binding cassette) and CaMdr1p (major facilitator superfamily) as reviewed in Ref. (13).

Due to the increasing resistance, several groups of researchers focus on safer and effective new antifungal compounds. Authors have isolated *Paracoccidioides* spp. (14) and *Candida* (15) susceptible to curcumin. The use of ajoene derived from garlic with antifungal activity against *Paracoccidioides brasiliensis* (16), *Scedosporium prolificans* (17), and dermatophytes (18) has also been reported. Antiretroviral protease inhibitors such as Saquinavir and Ritonavir have shown inhibitory activity against *Histoplasma capsulatum* (19) and *Candida albicans* (20). In addition, several other reports showing the antifungal activities of different compounds with potential use in patients have appeared, still without clearance from regulatory institutions.

Generally, the immune system is important to achieve good therapeutic results in association with antifungal drugs. The status of innate and adaptive immune system plays a central role in the protection against foreign pathogens. In contrast to immunocompetent individuals, immunosuppressed patients are much more susceptible to fungal infections some of them fatal (21, 22).

The cellular immune system is essential to protect and eliminate fungal pathogens; in general, dendritic cells (DCs), macrophages, and neutrophils are central in the mechanisms of fungal elimination. Antigenic peptides are presented to lymphocytes with subsequent eliciting of T-cell and B-cell effective responses (21–24). Differentiation of CD4^+^ T cells along a T-helper (Th) cell type 1 (Th1) or type 2 (Th2) pathway and development of specific Th responses determine host’s susceptibility or resistance to invasive fungal infections. A Th1 response is induced by cytokines, such as IFN-γ, interleukin (IL)-6, tumor necrosis factor (TNF)-α, and IL-12. The main Th2 cytokines are IL-4 and IL-10. IFN-γ activates macrophages and increases fungistatic and fungicidal activities. Th17 cells and IL-22 are involved in the activation and repair of epithelial barriers, and while activated by IL-17 are crucial for antifungal defense and control of the NK cells (21–25).

The function of antibody-mediated immunity against fungal infections was believed to have little or no role in protection against fungal diseases in the past (26). However, since Dromer et al. showed that a monoclonal antibody to *Cryptococcus neoformans* was effective against the fungal infection (27), a series of protective monoclonal antibodies against medically important fungi have been described (26). The protective mechanism of antibody-mediated immunity depends on opsonization, Fc receptor-dependent ADCC, immunoglobulin subclasses, genetic background, status of the cellular immune system, fungal burden, amount of patient administered monoclonal antibodies, among other characteristics (26).

Antifungal drugs are the basis of systemic mycoses treatment in both immunocompetent and immunosuppressed patients. However, immunosuppression or anergy may interfere with chemotherapy efficiency. Vaccination (therapeutic or prophylactic) may boost the immune system and add to the protective effect of antifungal drugs allowing for reduction of the time of treatment and prevention of relapse. In this review, we focus mainly on vaccines and epitope description.

## *Paracoccidioides brasiliensis* 

The major diagnostic antigen of *Paracoccidioides brasiliensis* is the 43 kDa glycoprotein (gp43) discovered in 1986 by Puccia et al. (28). A detailed description of gp43 was reviewed in Travassos et al. (29). Epitopes in the gp43 are peptide in nature so that patients’ sera reacted with the deglycosylated antigen (30). Several mAbs were raised to the gp43 and tested either *in vivo* against lung infections by *Paracoccidioides brasiliensis* or in phagocytosis assays with peritoneal and alveolar macrophages. Most mAbs stimulated the phagocytosis of yeast forms (31). MAb 3E, which was effective both in the reduction of fungal burdens in infected animals and in the promotion of phagocytosis, was tested for binding to a panel of gp43 internal peptides. The mAb 3E epitope lied within the sequence NHVRIPIGYWAV shared with *A. fumigatus, Aspergillus oryzae*, and *Blumeria graminis* sequences from β-1,3-glucanases (29).

Other protein antigens eliciting protective antibodies have been described but the B-cell epitopes were not characterized. In relation to the recently recognized *P*. *lutzii* species, the gp43 and 27 kDa antigens were less expressed in *P*. *lutzii* and PS2 genotype of *Paracoccidioides brasiliensis* (32). The gp43 ortholog in *P*. *lutzii* contains few epitopes in common with the *Paracoccidioides brasiliensis* gp43, contributing to serological diagnostic difficulties in patients infected with *P*. *lutzii* (33).

The gp43 elicits an IFN-γ-mediated T-CD4^+^ response, which is protective against the lung infection by *Paracoccidioides brasiliensis*. The gp43 was cloned, sequenced, and expressed in bacteria as a recombinant fusion protein (34). The amino acid sequence was deduced from a 987-bp fragment obtained by PCR amplification. Similarities of 56–58% were found with exo-1,3-β-d-glucanases of *Saccharomyces cerevisiae* and *Candida albicans*. The open-reading frame was found in a 1,329 bp fragment, encompassing two exons and one intron. The gp43 gene encodes 416 amino acids with a leader peptide of 35 aa. Epitopes able to elicit hypersensitivity in both guinea pigs (35) and humans (36) were described. A peptide of 15 amino acids (QTLIAIHTLAIRYAN) obtained from a collection of gp43 internal peptides, which was also located using DNAStar, Protean analysis Sette algorithm for Ia^d^ binding peptides (37), contained the T-CD4^+^ epitope, and was called P10 (38). The functional activities of P10 analogs and truncated peptides were studied. Peptides of 12 aa or longer, which is the size required for MHC II antigen presentation, were active. The sequence HTLAIR is an essential domain of the epitope. Gene polymorphism studies showed that the P10 sequence is highly conserved in *Paracoccidioides brasiliensis* isolates (39). In contrast, the corresponding sequences of *Candida albicans* and *S cerevisiae* exo-glucanases differed from P10 (40).

As with the gp43, P10 induces a Th1 lymphocyte response, which is protective against the intratracheal (i.t.) infection by virulent *Paracoccidioides brasiliensis*. IFN-γ is a key cytokine in this response as it has been shown to activate macrophages for increased fungicidal activity against *Paracoccidioides brasiliensis* and *Blastomyces dermatitidis* (41). It also plays a role in the organization of granulomas. Mice deficient in the IFN-γ are highly susceptible to *Paracoccidioides brasiliensis* infection. IFN-γ-receptor (but not IFN-α-R and IFN-β-R), IFN-γ, and IRF-1 KO mice were 100% killed 3–4 weeks following i.t. infection with virulent *Paracoccidioides brasiliensis*. P10 failed to protect those KO mice (40).

## P10 as a Vaccine Candidate

P10 contains the T-CD4^+^ epitope that is presented by MHC II molecules from murine H-2 haptotypes a, b, and d (38). The promiscuous nature of P10, if also shown with HLA-DR molecules, could represent an important attribute of this peptide to be used in a human vaccine to paracoccidioidomycosis. Iwai et al. (42) tested P10 and the analogous peptide gp43 (180–194), which included an N-terminal lysine and omitted the C-terminal asparagine, a glycosylated residue in the gp43. Both peptides bound to the nine prevalent HLA-DR molecules confirming their ability to be presented by different MHC II antigens. Gp43 (180–194) and four other peptides identified by TEPITOPE algorithm were recognized by 53 and 32–47%, respectively, of patients with treated paracoccidioidomycosis. Seventy-four percent of patients recognized a combination of five promiscuous gp43 peptides. TEPITOPE scanned 25 Caucasian HLA-DR antigens with P10 and analogous peptides, all containing the HTLAIR core sequence, being predicted to bind to 90% of them. The four peptides that were predicted to bind to a large number of HLA-DR molecules, in addition to Gp43 (180–194), were Gp43 (45–59): IGGWLLLEPWISPSV; Gp43 (94–108): TEDDFKNIAAAGLNHV; Gp43 (106–120): LNHVRIPIGYWAVNP; and Gp43 (283–298): IDQHVKLACSLPHGRL (42). These peptides could be added to P10 or Gp43 (180–194) in case the single epitope-based vaccine may not be powerful enough to induce full protective immunity. Indeed, multiple B-cell and T-cell epitopes in a pool or as a multi-epitope polypeptide were reported to increase immunogenicity (43, 44).

**Figure 1:**
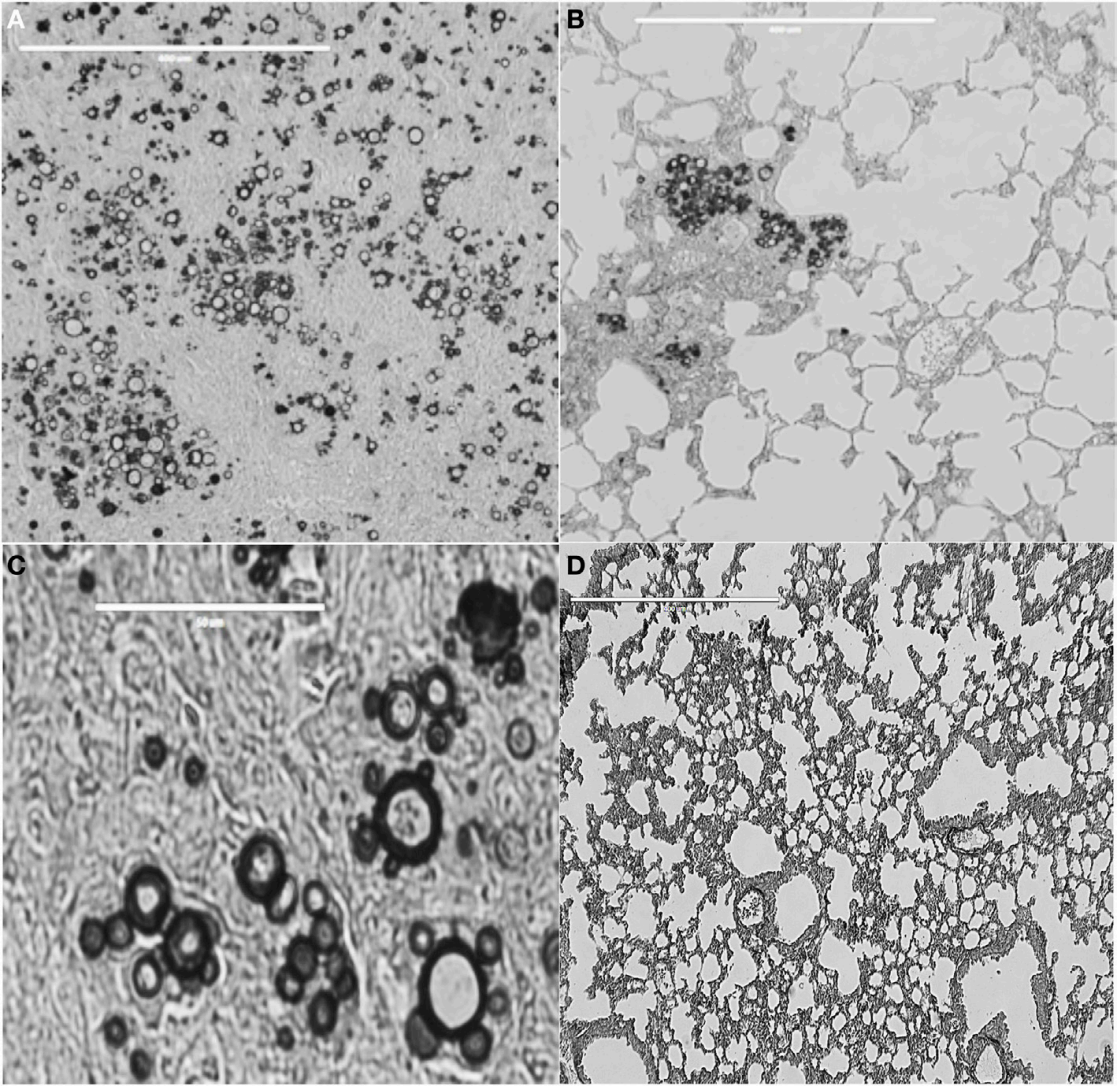
**Lung tissue from BALB/c mice infected with *Paracoccidioides brasiliensis* vaccinated or not with peptide P10**. **(A)** Lung tissue of control only infected mice. Bar, 400 μm. **(B)** Lung tissue of mice infected with *Paracoccidioides brasiliensis* and vaccinated with P10 in presence of cationic lipid. Bar, 400 μm. **(C)** Highly magnified lung tissue of control, only infected mice. Bar, 50 μm. **(D)** Lung tissue of mice infected with *Paracoccidioides brasiliensis* and vaccinated with P10 in presence of cationic lipid. Bar, 400 μm. Slides were stained with Grocott-Gomori methenamine silver. Pictures were taken using EVOS fluorescence microscopy (AMG). The Animal Care and Use Committee of the University of São Paulo approved all *in vivo* testing.

In the mouse experimental infection, P10 alone exerted an efficient antifungal immunity against i.t. infections by virulent *Paracoccidioides brasiliensis* strains (Figure 1). A classical demonstration by histopathology shows murine lung sections from i.t.-infected BALB/c mice with large granulomas and numerous fungal cells as compared to preserved lung parenchyma, few or no detectable granulomas, very few or absence of fungal elements, in P10 immunized mice (45). Early experiments used complete Freund adjuvant as an adjuvant to both gp43 and P10. Mayorga et al. (46) showed that mice treated with the cationic lipid dioctadecyl-dimethylammonium bromide (DODAB) followed in efficiency by bacterial flagellin, both adjuvants to P10, were best protected against fungal infection as demonstrated by the lowest numbers of viable yeast cells and reduced granuloma formation and fibrosis. IFN-γ and TNF-α, in contrast to IL-4 and IL-10, were secreted in the lungs of mice immunized with P10 in combination with these adjuvants. When combined with antifungal drugs, P10 was protective even in animals submitted to severe immune suppression (47). P10 immunization together with itraconazole or sulfamethoxazole and trimethoprim chemotherapy resulted in 100% survival of infected immunocompromised mice, up to 200 days postinfection, whereas untreated anergic mice died within 80 days.

P10 is primarily an antigenic peptide that is presented by MHC II molecules to induce a Th1 T-CD4^+^ cell proliferation, which exerts an IFN-γ-dependent antifungal protection. Generally, T-CD4^+^ cells confer resistance through secretion of cytokines such as IFN-γ, TNF-α, GM-CSF, and IL-17A, which can activate neutrophils, macrophages, DCs, and inflammatory monocytes for fungal killing and clearance. Activation of B cells leads to the secretion of protective antibodies (22, 48).

There is, however, some evidence of an immunomodulatory effect of P10 *in vivo*, which parallels the biological effects of isolated immunoglobulin CDRs and fragments of transcription factors (49, 50). These short peptides were able to activate bone-marrow DCs, which in turn started an immune response protective against antigenically unrelated metastatic murine melanoma.

A similar effect was observed with the combination of P10 and the TLR-5-binding *Salmonella typhimurium* FliCi flagellin (51), using the same metastatic melanoma system. Compounds were administered intranasally into C57Bl/6 mice, challenged intravenous (i.v.) with syngeneic B16F10-Nex2 murine melanoma cells. A marked reduction in the number of pulmonary tumor cell nodules was observed with a significant increase in the survival of challenged animals. Noticeable immunological responses were the M1 lung macrophages and secretion by lymph node cells and splenocytes of IL-12p40 and IFN-γ when they were restimulated with tumor antigens.

Therefore, P10 acts not only as a specific Th1 *Paracoccidioides brasiliensis* antigen but also as a non-specific immunomodulatory peptide, much like a series of other anti-cancer peptides (49).

*Ex vivo* P10-primed bone-marrow DCs were administered to *Paracoccidioides brasiliensis* i.t.-infected mice (52). There followed a significantly reduced fungal burden and decreased pulmonary damage. Increased production of IFN-γ and IL-12 and reduction in IL-10 and IL-4 compared to the untreated or unprimed, DCs-treated mice were obtained. A vaccine, therefore, with P10-primed DCs has the potential of rapid protection against the development of serious paracoccidioidomycosis in infected patients.

## P10 Mini-Gene Therapy

An early plasmid vaccine with a mammalian expression vector carrying the gp43 gene induced specific antifungal antibodies and a T cell-mediated immune response under the control of the CMV promoter (53). The IFN-γ-mediated immune response, which was effective against the i.t. infection by *Paracoccidioides brasiliensis*, lasted for at least 6 months after DNA-vaccine administration. Plasmids with P10 mini-gene insert (pP10) and also with IL-12 insert (pIL-12) were later used in an immunoprophylactic protocol and their association completely eliminated the fungal elements [colony forming units (CFUs)] in the lungs from i.t.-infected animals (54). In a therapeutic protocol, empty plasmids were inactive and only the combination of both pP10 and pIL-12 achieved maximal protection using both BALB/c and B10.A (susceptible) mouse strains.

In a long-term protocol in which plasmids were administered in B10.A susceptible mice, 30 days after infection and the animals were sacrificed 6 months after infection, the pP10 vaccine alone reduced lung CFUs more than 100-fold and the combination of pP10 and pIL-12, virtually eliminated all fungal cells with recovery of the lung architecture. These are very encouraging results toward the use of gene therapy with P10 DNA insert, along with pIL-12 for a long lasting immune protection against paracoccidioidomycosis.

The above-described results are a remarkable example of effective immune responses elicited by a single epitope against a systemic fungal infection. Their complexity, however, is far from being completely understood. Owing to persistent antigen stimulation and active immune response, the infection by *Paracoccidioides brasiliensis* is characterized by granuloma formation and fibrosis. Remarkably, the association of the cationic lipid (DODAB) and P10 resulted in significant reduction of pulmonary fibrosis in animals developing paracoccidioidomycosis.

## Other Agents of Systemic Mycoses

### *Coccidioides immitis* and *Coccidioides posadasii*

As with *Paracoccidioides brasiliensis*, T cell-mediated immunity seems to be most important in the protection against *Coccidioides* infection (55–57). High titers of antibodies correlate with poor clinical prognosis, although there is evidence showing that a specific humoral response can modulate the immune response and contribute to host resistance (55–57).

A cell wall associated proline-rich antigen known as antigen 2 (Ag2) and Ag2/Pra showed to be protective against *Coccidioides* infection using an experimental model (58, 59). A recombinant rAg2/Pra protein and a genetic vaccine with AG@/PRA elicited protective CD4^+^ T-cell-mediated response, although the route of immunization with both antigens showed some inconsistence (59).

Herr et al. (59) showed that *Coccidioides posadasii* produces a homologous proline-rich antigen denominated Prp2, which shows 69% protein identity and 86% similarity to Ag2/Pra. Protection against intra nasal challenge of C57BL/6 mice was verified by subcutaneous vaccination with single bacterially expressed homolog, rAg2/Pra or rPrp2 in association with rAg2/Pra in the presence of the CpG oligodeoxynucleotides adjuvant (59). A significant improvement of protective immunity induced by vaccination with combined rAg2/Pra and rPrp2 proteins was observed when compared to immunization with the single recombinant proteins (59).

Peptide libraries from proline-rich Ag2/Pra and Prp2 were used for mapping CD4^+^ T-cell epitope by analysis of the T-cell response in an IFN-γ-ELISPOT assay. Six sequences of Ag2/Pra overlapping peptides (TRLTDFKCHCSKPELPGQIT, HCSKPELPGQITPCVEEACP, PIDIPPVDTTAAPEPSETAE, TTAAPEPSETAEPTAEPTEE, PTEEPTAEPTAEPTAEPTHE, and PTAEPTAEPTHEPTEEPTAV) and three sequences of PrP2 (EKLTDFKCHCAKPELPGKIT, DTRTPTQPPSTSPSAPQPTA, and PSTSPSAPQPTACI-PKRRRA) induced IFN-γ by CD4^+^ T cells isolated from mice immunized with either rAg2/Pra or rPrp2 (59). Albeit some peptides exhibited high similarity in their sequences, cross-reactions with T cells from either rAg2/Pra or rPrp2-immunized mice were not observed. Peptide sequences with high T-cell stimulatory response from homologous immunized mice contained one or more TXX’P sequences. The XX residues, however, of TXX’P motifs of Ag2/Pra and Prp2 differed (59).

Hurtgen et al. (60) described a strategy for the construction and immunological evaluation of a recombinant epitope-based vaccine. The use of a computational algorithm (ProPred), which identified putative T-cell epitopes predicted to bind promiscuously to human MHC class II molecules, revealed three antigens: aspartyl protease (Pep1), alpha-mannosidase (Amn1), and phospholipidase B as potential vaccine candidates (60). T-cell reactivity of synthetic peptides carrying all predicted epitopes was tested by IFN-γ ELISPOT assay.

A single, bacteria-expressed, and recombinant epitope-based vaccine was constructed with five promiscuous, immunodominant T-cell epitopes derived from Pep1 (MRNSILLAATVLLGCTSAKVHL and HVRALGQKYFGSLPSSQQQTV), Amn1 (PAKVDVLLAQSLKLADVLKF and NGLATTGTLVLEWTRLSDIT), and P1b (TPLVVYIPNYPYTTWSNIST). The upstream 20-mer peptide had the N-terminal of each epitope flanked by Ii-Key fragment (LRMKLPKS), and the C termini in four of the five peptides were flanked by CPGPG spacer to avoid processing of junctional epitopes (60).

C57BL/6 mice immunized with the epitope-based vaccine admixed with synthetic CpG ODN adjuvant or loaded on yeast glucan particles, and then challenged intranasally with *Coccidioides posadasii*, induced an infiltration of active T helper-1 (Th1), Th2 and Th17 cells, enhanced IFN-γ and IL-17, and reduced lung fungal burden with prolonged animal survival (60).

In some infections by dimorphic fungi, even in the absence of CD4^+^ T cells, mice had long-term survival mediated by vaccine-induced IL-17-producing CD8^+^ T cells (61). Recombinant CD4^+^ and CD8^+^ T-cell epitopes joined by non-immunogenic linkers were loaded on glucan particles (composed primarily of β-1,3-glucan) which delivered the vaccine to APCs. Beta-glucan activates the alternative pathway of complement with deposition of C3 fragments, thus leading to phagocytosis by DCs and macrophages mediated by complement receptors and dectin-1. A decapeptide (EP67) agonist of active C-terminal region of human complement C5a acted as an adjuvant enhancing antigen presentation by macrophages and DCs but not neutrophils due to its high affinity for C5a receptors (C5aR/CD88) (61). This adjuvant was effective when conjugated with lysine residues on the surface of live arthroconidia from the vaccine strain. EP67 directs the vaccine to C5aR-bearing macrophages and DCs, inducing phagocytosis and antigen presentation. BALB/c mice immunized with EP67 conjugated, live vaccine, increased survival and decreased inflammatory pathology, fungal burden, and neutrophils in the lungs (62). EP67 conjugated with epitope-based protein vaccines may provide an effective mechanism to further augment Th17 immunity (61). The use of glucan particles as a delivery and adjuvant compound as used here to treat coccidiomycosis could become in the future an important carrier of peptide antigens eliciting protective immune cellular responses, thus following the pioneering work in *Candida albicans*. Coincidently with *Candida albicans*, the cellular response elicited against *Coccidioides* was also characterized by increased Th17 immunity.

### *Histoplasma capsulatum* 

Scheckelhoff and Deepe described an immunogenic heat shock protein-60 region (F3, fragment 3), which conferred protection against experimental *Histoplasma* infection (63, 64). A T-cell clone from C57BL/6 mice expressing Vβ 8.1/8.2^+^ T cells was generated after subcutaneous rHsp60 immunization and was efficacious for rHsp60-induced protective effect. TCR analysis showed that a subset of Vβ 8.1/8.2^+^ that produced IFN-γ and reacted with F3, shared a common CDR3 sequence, DGGQG (64). It seems that a distinct subset of Vβ 8.1/8.2^+^ T cells is crucial for generating a protective response following rHsp60 immunization.

CD4^+^ T-cell depletion during primary infection by *H. capsulatum* led to animal’s death, whereas lack of CD8^+^ T cells decreased fungal clearance (65). Remarkably, however, CD4^+^ T cells are dispensable in vaccine immunity to *H. capsulatum* [reviewed in Ref. (66)]. In the absence of CD4^+^ T cells, CD8^+^ T cells must be present exclusively during vaccine induction. Alternatively, immune CD8^+^ T cells generated in wild type mice, in the absence of CD4^+^ T cells, were adoptively transferred to mice infected with *Blastomyces* giving rise to effector cells lowering by 15-fold the lung CFU compared to no T cells. In both *H. capsulatum* and *B. dermatitidis* infections, when CD4^+^ T cells are absent, CD8^+^ T cells participate as effectors of vaccine immunity against these fungi (67). Likely, MHC-I molecules cross-present exogenous fungal antigens to vaccine-induced CD8^+^ T cells. These results point to the feasibility of developing vaccines against fungal infections in patients with immune deficiencies such as AIDS. They also illustrate the plasticity of the immune system adding unsuspected functional roles to cells and soluble mediators.

### *Aspergillus fumigatus* 

Invasive aspergillosis has significant incidence in immunocompromised hosts, with high mortality rate. Ito et al. demonstrated that sonication of *A. fumigatus* hyphae liberated an antigen able to protect corticosteroid immunosuppressed mice from invasive aspergillosis (68). Subcutaneous vaccination with recombinant allergen Asp f3, a 19 kDa protein recognized by antibodies from mice exposed intranasally to *A. fumigatus* conidia, with or without TiterMax adjuvant was protective (68). Two T-cell epitopes have been identified, and orthologs of Asp f3 have also been found in other *Aspergillus* species, *Coccidioides posadasii, Penicillium citrinum, Candida albicans, Candida boidinii, S. cerevisiae*. Since Asp f3 could mediate allergic bronchopulmonary aspergillosis, authors focused on eliminating its allergenic property after mapping the reactive epitopes. Several truncated forms of Asp f3 were synthesized and by using mass spectrometric analysis, two peptides were identified, 11-mer (PGAFTPVCSAR) and 13-mer (HVPEYIEKLPEIR), able to stimulate Asp f3-specific T cells (68).

The protection mediated by Asp f3 was investigated in experimentally infected mice. After vaccination, specific Asp f3 pre-infection IgG titers did no differ in resistant and susceptible mice and passive transfer of Asp f3 antibodies did not protect immunosuppressed mice from aspergillosis. In fact, the antigen is not accessible unless both cell walls and membrane have been permeabilized (69). Depletion of CD4^+^ T cells, however, reduced the survival of rAsp f3-vaccinated mice. Transference of purified CD4^+^ T cells from rAsp f3-vaccinated mice into non-vaccinated mice conferred protection (69).

Consecutive 5-aa overlapping peptides from Asp f3 (15–168) sequence were synthesized. Mice were vaccinated subcutaneously with non-allergenic recombinant Asp f3 (15–168)-based vaccine, suspended in TiterMax adjuvant. Five weeks after the second immunization, mice were immunosuppressed with subcutaneous injection of cortisone acetate (2.5 mg) in suspension with methylcellulose (0.5%) and Tween 80 (0.1%) for 10 days. Mice were then anesthetized and intranasally inoculated with three million conidia. Significant protection was observed with such rAsp f3 vaccination (69).

Diaz-Arevalo et al. refined the previous search for immunogenic Asp f3 epitopes (70). T-cell proliferation with a set of overlapping synthetic 20-mer peptides was carried out. T cells from Asp f3 (15–168)-vaccinated non-infected mice as well as vaccinated infected survivors showed proliferative responses to the synthetic peptides: VCSARHVPEYIEKLPEIRAK (residues 60–79) and EIRAKGVDVVAVLAYNDAYV (residues 75–94). Sera from vaccinated survivors of experimental *A. fumigatus* challenge and from non-surviving mice were analyzed. Elevated titers of IgG to VCSARHVPEYIEKLPEIRAK were found only in the surviving group suggesting that the deduced sequence contains both a B-cell epitope and a T-cell epitope (70).

Vaccination of a susceptible population to an opportunistic disease like invasive aspergillosis was approached by Stevens et al. (56). The least immunocompromised patients might be considered as an initial step. Candidates to immunization could include chronic granulomatous disease patients, transplant, leukemics, solid tumor at diagnosis, rheumatic or inflammatory bowel, and intensive care unit patients. Donors of hematopoietic stem cell transplants are also immunization candidates. As mentioned above, CD8^+^ T cells can be used in CD4-deficient hosts, and vaccines can be used aiming at stimulating the immune response, reducing immunosuppression, or acting synergistically with antifungal therapy.

### *Candida albicans* 

Invasive candidiasis is often associated with immunosuppression, prolonged antibiotic treatment, and anatomical lesions like surgery or venous catheter. A mortality of >30% is observed. Other clinical forms of candidiasis such as skin infections, oropharyngeal mucosa, and vaginal are most frequent but less severe. Knowledge of protective immune responses in candidiasis is thus a major aspect to be pursued in the field of systemic mycoses. Bär et al. used immunoproteomics to investigate natural T-cell epitopes of *Candida albicans*. The authors identified an MHC II-bound peptide that is recognized by 1/4 of all *Candida albicans*-specific Th cells, a remarkably high frequency of interaction (71).

Four peptides were identified, with overlapping sequences, derived from a homologous region of the related adhesins Als1 and Als3. The longest of the three identified Als1/Als3-derived peptides (amino acid residues 236–253) was chosen for further analysis, referred to as pALS [sequences, indicating in bold the predicted MHC II-binding epitope: KGLND**WNYPVSSES**FS(Y)(T)]. The novel antigenic peptide of Bär et al. has an important role in fungal pathogenicity. It is functionally conserved in non-albicans *Candida* species, and most importantly, the epitope-specific T cells are not only murine but also human. Human memory Th cells responded to peptide stimulation, and vaccination of mice with the peptide elicited a T cell-dependent anti-candidiasis immune response (71). The pALS peptide of *Candida albicans*, carrying a promiscuous epitope, and eliciting a protective antifungal immune response, is functionally similar to *Paracoccidioides brasiliensis* P10, a peptide candidate of a vaccine against systemic paracoccidioidomycosis (see above section). pALS-specific T cells from the cervical lymph nodes of orally infected mice secreted IL-17A, but not IFN-γ or IL-4 (71).

Another methodology was used by Wang et al. (72), who evaluated a hybrid phage as a potential vaccine candidate without adjuvant against *Candida albicans*. The ability of hybrid phage displaying epitope SLAQVKYTSASSI to induce an immune protective response was studied in a mouse model. Strong cellular and humoral immune responses were induced similar to recombinant r*Sap*2 protein immunization. Protection against intravenous lethal challenge with *Candida albicans* was observed in BALB/c mice immunized with hybrid phage confirming its great potential as a vaccine inducing strong Th1 and Th17 response without adjuvant (72).

A vaccine that could be effective against *Candida albicans* and a variety of other human pathogenic fungi was proposed by Cassone and coworkers (73, 74), based on *Laminaria digitata*’s β-glucan (laminarin). To increase the immunogenicity of the glucan, it was conjugated with the diphtheria toxoid CRM197. The conjugate was protective against systemic and vaginal *Candida albicans* infections, by eliciting anti-β-glucan antibodies, mainly IgG2b. These antibodies bound to and inhibited growth of both *Candida albicans* and *A. fumigatus* hyphae.

To understand the nature of the epitopes recognized by protective antibodies to these conjugates, studies were carried out with the following compounds, in addition to the laminarin-diphtheria toxoid (CRM197) conjugate, which was protective against fungal infections in mice (75): natural curdlan (Curd)-CRM197; linear 15-mer-CRM197 or 1,6-branched-17-mer-CRM197 β-(1,3)-glucan-derived oligosaccharides. Anti-β-(1,3)-glucan IgG antibodies were specifically raised by Curd-CRM197 and 15-mer-CRM197 oligosaccharide immunization. These antibodies protected mice against lethal infection by *Candida albicans*. Contrariwise, immunization with the 1,6-branched-17-mer CRM197 oligosaccharide elicited both anti-β-(1,6) and anti-β-(1,3) glucan IgG, which was not protective (75).

Conjugation of β-glucan to a carrier protein induces the production of antibodies that are protective against major fungal pathogens such as *Aspergillus* spp. and *Cryptococcus* spp., in addition to *Candida* spp. Growth-inhibitory β-glucan-specific antibodies combined with a protein, such as Als3 or Hyr1, could enhance the magnitude of protective antibodies as well as reduce the chances of *Candida albicans* immune evasion (76). The importance of a multivalent vaccine in comparison with the univalent anti-β-glucan-specific antibodies (42) was further evaluated by using mixed pALS with curdlan for protective immunization. Mice were challenged 3 weeks later i.v., with a high dose of *Candida albicans*. Immunization with curdlan alone was not sufficient for protection but the combination with pALS greatly increased the number of mice protected from fatal systemic candidiasis. The enhanced survival upon immunization with pALS plus curdlan correlated with the induction of pALS-specific IL-17A-producing CD4^+^ T cells. Data show therefore that pALS-specific Th17 lymphocytes do protect mice from candidiasis (71).

On Table 1, we summarize the linear peptides carrying epitopes potentially effective in antifungal vaccine development.

**Table 1:** **Linear peptide sequences with potential use as vaccine components**.

Fungi (reference)	Name of antigen and linear peptide sequencie	Immune cell	Animal model delivery adjuvancy	Results
*Paracoccidioides brasiliensis* (38, 46, 52, 54)	P10	CD4^+^ Th1 cell	BALB/c mice/CFA, alumen, CL, flagellin, DC, DNA plasmid	Protection against i.t. challenge, reduction of fungal burden, efficacy of DNA vaccine
	QTLIAIHTLAIRYAN			

*Coccidioides* spp. (59)	Antigen 2 (Ag2)/Pra	CD4^+^ Th1 cell	C57BL/6 mice/CpG ODN	Elicit T-cell response in mice immunized with rAg2/Pra; IFN-γ ELISPOT
	1P6: TRLTDFKCHCSKPELPGQIT; 1P7: HCSKPELPGQITPCVEEACP; 1P12: PIDIPPVDTTAAPE-PSETAE; 1P13: TTAAPEPSETAEPTAEPTEE; 1P15: PTEEPTAEPTAEPTAEPTHE; 1P16: PTAEPTAEPTHEPTEEPTAV			

*Coccidioides* spp. (59)	PrP2	CD4^+^ Th1 cell	C57BL/6 mice/CpG ODN	Elicit T-cell response in mice immunized with rPrP2; IFN-γ ELISPOT
	2P6: EKLTDFKCHCAKPELPGKIT; 2P13: DTRTPTQPPSTSPSAPQPTA; 2P14: PSTSPSAPQPTACI-PKRRRA			

*Coccidioides* spp. (60)	Predicted T-cell epitopes Pep1	CD4^+^ T cells	HLA-DR4 C57BL/6 mice/CpG ODN	Elicit T-cell response in mice immunized with rEBV; IFN-γ ELISPOT
	P1: MRNSILLAATVLLGCTSAKVHL; P2: HVRALGQKYFGSLPSSQQQTV			

*Coccidioides* spp. (60)	Predicted T-cell epitopes Amn1	CD4^+^ T cells	HLA-DR4 C57BL/6 mice/CpG ODN	Elicit T-cell response in mice immunized with rEBV; IFN-γ ELISPOT
	P10: PAKVDVLLAQSLKLADVLKF; P11: NGLATTGTLVLEWTRLSDIT			

*Coccidioides* spp. (60)	Predicted T-cell epitopes phospholipidase B (Plb)	None	HLA-DR4 C57BL/6 mice/CpG ODN	Failed to elicit T-cell response from mice immunized with rEBV; IFN-γ ELISPOT
	P6: TPLVVYIPNYPYTTWSNIST			

*Coccidioides* spp. (60)	Recombinant epitope-based vaccine rEBV	CD4^+^ Th1, Th2 and Th17 cells	HLA-DR4 C57BL/6 mice/CpG ODN or GPs plus OVA complex	(a) *In vitro* T-cell response in mice immunized with rEBV; IFN-γ ELISPOT. (b) rEBV + CpG ODN and i.n. challenge: reduction of lung CFU but not significant survival. (c) rEBV + GPs: 10-fold-higher T-cell response with Pep1-P1 and significant enhanced survival.
	Include the five selected epitope peptides (Pep1, Amn1 and Plb), N-terminal leader peptides and glycine/proline spacer sequences (CPGPG)			

*Histoplasma capsulatum* (64)	CDR3 fragment	Vβ 8.1/8.2^+^ T cells	C57BL/6 and athymic nude mice/TCR α/β^–/–^ and IFN-γ–/– mice	(a) rHsp60 or fragment 3 (F3) confers protection after i.n. challenge. (b) Depletion of Vβ 8.1/8.2^+^ T cells from immunized rHsp60 mice abolish the protection to lethal and sublethal challenges.
	DGGQG			

*Aspergillus* spp. (68, 78)	Asp f3	T cell	CF-1 mice/TiterMax	(a) rASP f3 confer protection to corticosteroid immunosuppressed CF-1 mice against i.n. infection with conidia. (b) T-cell proliferation to rAsp f3 variants and trypsin-derived peptides (B12 and C3) in immunized CF-1 mice.
	B12: PGAFTPVCSAR, C3: HVPEYIEKLPEIR			

*Aspergillus* spp. (68, 70)	Asp f3	B and T cells	CF-1 mice/TiterMax	(a) CD4^+^ T cells are required for rAsp f3 vaccine protection. (b) Proliferation of T cells from rAsp f3-vaccinated mice and tested by luminometric ATP cell titer quantification in positively selected T cells after stimulation. (c) P4: VCSARHVPEYIEKLPEIRAK IgG titers were elevated only in the surviving vaccinated and *Aspergillus fumigatus* challenged mice.
	P4: VCSARHVPEYIEKLPEIRAK; P5: EIRAKGVDVVAVLAYNDAYVVCSAR			

*Candida albicans* (71)	pALS (ALS1, ALS3)	CD4^+^ Th17 cell	C57BL/6 and JHT mice/IFA mixed with curdlan or CpG	(a) Peptide-loaded MHCII complex from DC1940 cells isolated and sequenced by liquid chromatography coupled to MS/MS. (b) Mice immunized with pALS mixed with IFA plus curdlan and i.v. infected with *Candida albicans* protected from fatal systemic disease. (c) pALS is recognized by human memory T cells.
	KGLND**WNYPVSSES**FS(Y)(T)			

*Candida albicans* (72)	Hybrid phage displaying epitope	B and T cells	BALB/c mice/TE buffer or CFA	(a) Decreased colonization of *Candida albicans* in kidneys and spleens from mice immunized with hybrid phage (TE) or rSap2 (CFA). (b) Mice immunized with hybrid phage (TE) or rSap2 (CFA) prolong survival against *Candida albicans* infection.
	SLAQVKYTSASSI			
	Recombinant Sap2 (rSap2)			

Pan fungal (77)	Calnexin peptide #1	CD4^+^ Th1 and Th17 cells	C57BL/6/GP-MSA and yeast RNA; LPS	(a) Calnexin (ER protein) has *Blastomyces dermatitidis, H. capsulatum, Coccidioides posadasii*, and *Paracoccidioides brasiliensis* conserved regions. (b) Immunized mice with rCalnexin formulated in GP reduced lung and spleen CFU in mice infected with *B. dermatitidis* or *Coccidioides posadasii* and prolonged survival. (c) Soluble calnexin peptide #1 plus LPS delivery by i.v. route improved expansion of calnexin-specific T cells.
	LVVKNPAAHHAIS			
	Recombinant calnexin (rCalnexin)			

*CFA, complete Freund adjuvant; IFA, incomplete Freund adjuvant; CL, cationic lipid; DC, dendritic cells; CpG ODN, synthetic oligodeoxynucleotide containing unmethylated CpG dinucleotides; i.t., intratracheal; i.n., intransal; i.v., intravenous; rEBV, bacterium-expressed recombinant epitope-based vaccine; GPs, yeast cell wall-derived glucan particles; GMP, glucan mannan particles; CFU, colony forming unit; OVA, chicken ovalbumin; MSA, mouse serum albumin*.

## Pan-Fungal Vaccines

Recent studies showed that attenuated *Blastomyces dermatitidis* conferred protective effects by T-cell recognition of an unknown but conserved antigen [reviewed in Ref. (42)]. Wüthrich et al. using transgenic CD4^+^ T cells identified an amino acid determinant within chaperone calnexin that is conserved across ascomycetes (77). Calnexin, an ER protein, localizes to the surface of yeast, hyphae, and spores (77). Infection with dimorphic or opportunistic fungi induces calnexin-specific CD4^+^ T cells (77). Vaccine of calnexin in glucan particles elicited calnexin-specific CD4^+^ T cells and resistance to infection by *B. dermatitidis, H. capsulatum, Pseudogymnoascus* (*Geomyces*) *destructans, Fonsecaea pedrosoi*, and *A. fumigatus* (77). Authors investigated regions of conserved sequences, which represent shared epitopes recognized by the 1807-T cell receptor. Using an algorithm that predicts six regions of overlapping peptide and a second algorithm developed by Marc Jenkins refined the analysis (77). Peptides of 13-mer were synthesized, representing 10 predicted epitopes, and they were tested for binding to the 1807-T cell receptor. The peptide #1 (LVVKNPAAHHAIS) activated naive 1807-T cells as measured by their reduced expression of CD62L, increased expression of CD44, and stimulated production of IFN-γ. None of the other calnexin peptides induced IFN-γ production by 1807-T cells (77). To investigate the biological relevance of peptide #1 in medically important fungi with conserved calnexin sequences, naïve 1807-T cells were transferred into mice before infection or vaccination with these fungi (77). One week later, activation of 1807 and endogenous Ag-specific CD4^+^ T cells using calnexin peptide-MHC class II tetramer were analyzed. *B. dermatitidis, A. fumigatus, H. capsulatum, Coccidioides posadasii, F*. *pedrosoi*, and *Pseudogymnoascus* (*Geomyces*) *destructans* expanded and activated 1807 and tetramer-positive CD4^+^ T cells *in vivo*. Fungi that did not trigger expansion of tetramer-positive CD4^+^ T cells included *Candida albicans, Cryptococcus neoformans*, and *Pneumocystis jiroveci*, and any tetramer-positive CD8^+^ T cells detected in vaccinated mice (77). Vaccination with calnexin formulated in glucan particles or Adjuplex induces protective immunity against lethal, pulmonary fungal infection with *B. dermatitidis* and *Coccidioides posadasii*. Fungal burdens were reduced 10-fold in lung and spleen samples (77).

## Final Remarks

Albeit most single- or pan-antifungal vaccine in development focus on protein/peptide, live-attenuated fungi, immune stimulatory adjuvants, antigens presented by DCs, combination of polysaccharide with protein where polysaccharide acts as a carrier or as a mixing adjuvant, and on passive immunotherapy, the synthesis of linear oligosaccharides of β-glucan becomes also an alternative to a pan-fungal vaccine.

Liao et al. (78) developed a series of synthetic β-glucan oligosaccharides coupled to keyhole limpet hemocyanin (KLH) to generate glycoconjugates that contained structurally well-defined carbohydrate antigens. The authors have demonstrated, using a mouse model, that the conjugate of KLH and octa-β-glucan can elicit protective immune responses against *Candida albicans* (78).

Although short peptides carrying epitopes mediating immune responses may display remarkable activities, even acting as antigens and immunomodulatory molecules, it seems that multivalent vaccines may be superior to univalent ones, thus supporting Cassone’s views on *Candida albicans* vaccines (76). Development of an immune response against “multiple unrelated virulence traits” will probably be “a better approach.”

## Author Contributions

LT and CT wrote and revised the paper.

## Conflict of Interest Statement

The authors declare that the research was conducted in the absence of any commercial or financial relationships that could be construed as a potential conflict of interest.
